# Measuring the Wave Height Based on Binocular Cameras

**DOI:** 10.3390/s19061338

**Published:** 2019-03-17

**Authors:** Yan Cang, Hengxiang He, Yulong Qiao

**Affiliations:** College of Information and Communication, Harbin Engineering University, Harbin 150001, China; cangyan@hrbeu.edu.cn (Y.C.); qiaoyulong@hrbeu.edu.cn (Y.Q.)

**Keywords:** binocular stereo, wave height detection, micro-scale wave, stereo match

## Abstract

The wave is an important hydrological element in marine research. Accurately describing the characteristics of waves is therefore significant to the study of marine power. The contents of this article are as follows: (1) a wave height measurement system using binocular cameras is proposed, and the small tank experiments are conducted to prove the efficacy of the proposed system; (2) based on the scale invariant feature transition (SIFT) algorithm, sub-pixel Harris corners are calculated in the difference-of-Gaussian (DOG) space to locate key points more accurately; and (3) a bi-directional epipolar constraint is employed to decrease the mismatch rate and computation time.

## 1. Introduction

The waves contain abundant information. The extraction of useful wave information and its application to numerous fields has attracted increasing interest in marine research, including military research [1], industrial research [2], and meteorological research [3]. The wave detection parameters include the height, direction and waveform. Based on differences among the measurement methods, wave detection techniques can be classified into various types, such as aerometry, photogrammetric techniques [4], radar measurements [5], electronic measurements [6], optical measurements [7], and acoustic measurements [8]. The photogrammetric method is a non-contact measurement technique; this means that no sensors are launched onto the water surface, and the propagation of waves is not impacted. However, while photogrammetry is more convincing than radar detection and has a higher accuracy, it is still challenging to employ due to multiple complicated interfering factors, such as heavy illumination, optical rejection and refraction, and unobvious textural features on the water surface. Moreover, most existing photogrammetric approaches focus on monocular camera detection. It is hard to measure the real scales of the object from only one image. Either fractional laser or reference cameras are used when drawing the three-dimensional surface figure and contour map of the wave height [9,10]. The scale invariant feature transition (SIFT) algorithm is the best method for matching the image and reference image [11]. Accordingly, in this paper, we propose a binocular camera detection system to avoid the limitations of monocular detection. The proposed detection system synchronously captures left and right images and uses a stereo matching algorithm to extract key points that represent the textural characteristics of the wave and then reconstructs the wave surface and draws a wave contour map by using the rotation and translation matrices of binocular cameras. Specifically, we design a customized pattern and project it onto the water surface to enforce the textural features of the wave. In the stereo matching algorithm, we calculate sub-pixel Harris corners instead of extreme points in the difference-of-Gaussian (DOG) space of the SIFT vector to control the quantity of features by choosing different Harris corner response thresholds. In the matching process, we define the correct matching pairs that must be satisfied with bi-directional epipolar constraints.

## 2. Related Work

Based on the photogrammetric technique, wave measurements can be divided into three classes:

(1) Using a stereoscopic system and image correlation: Tsubaki [12] dyed water to make it a white colour and projected a random pattern on the surface; they also used particles as surface markers to reconstruct the water surface. In the same year, Douxchamps [13] used floating particles to strengthen the features of the water surface to measure the surface flow field. Felice [14] proposed an algorithm based on the particle image velocity (PIV) to measure tiny flows around a ship. In addition, Turney [15] developed a stereo system based on three-dimensional PIV (3D-PIV) measurements and measured the wave tunnel shape and velocity at the air–water interface. Fedele [16] introduced an epipolar constraint to decrease the search range and matching time by investigating the stereo matching component of the photogrammetric measurement technique. Viriyakija [17] proved that binocular stereo measurements work well at acquiring wave height measurements; the stereo photogrammetric measurement method agreed well with the traditional wave height metre in a tank. Chatellier [18] used image processing for the first time by using an edge detection algorithm to reconstruct the free surface shape and an image correlation algorithm to calculate the wave velocity; however, this method was highly dependent on the definitions of the camera parameters. Gomit [19,20] used two stereo systems to separately record an image correlation; one system used floating particles, whereas the other used moderate PIV particles, both of which can reconstruct small-scale ship models and perform reconstruction analyses of free surfaces accurately.

(2) Based on the properties of light penetration into a fluid: Suzuki [21] described the relation between light refraction at the bottom of a tank and near the surface of water; the light distribution was represented by a two-order differential equation that was calculated using the fast Fourier transform. In 2006, based on diffuse reflection at the surface, Sanada [22,23] proposed a refracted light distribution based on an image measurement technique. In 2010, Ng [24] measured the free surface and velocity vector fields by combining the visual reflection and PIV techniques. The PIV was used to explore the random pattern generated by the particles, and a reference pattern was placed beneath the water surface; therefore, the gradient profile can be estimated by comparing the reflection and reference patterns. However, such large-scale instruments require a faster algorithm.

(3) Based on the projection of a regular pattern: in 2008, Cochard [25] proposed an algorithm based on a stipple projection and applied it to acquire free wave measurements of overflow due to the breakage of a dam. Their method projects a deformed stipple onto the surface to reconstruct the free surface. To accurately record the change in the waveform, their paper explored a new system including a digital camera and synchronous projector that could measure an area of 1.8 × 1.1 m^2^. However, when the area of free surface is bigger, the edge of the projection pattern became obscure. The paper designs an irregular pattern to resolve this problem. In 2009, Cobelli [26] proposed an optical profile measurement technique that could measure the free surface deformation at each instant. The system consisted of a high-resolution video projection and a digital camera. The phase diagram was generated by calculating the difference between the deformed stripe pattern and the reference pattern. Compared with other methods, the advantage of the system was that it offered contactless measurements over large-scale areas; moreover, the measurement and analysis system could measure the free surface deformation with 6 × 106 sample points over a sampling area of 450 × 300 mm^2^ with a vertical resolution of 0.2 mm.

## 3. The Proposed Stereo Match Algorithm

**Figure 1:**
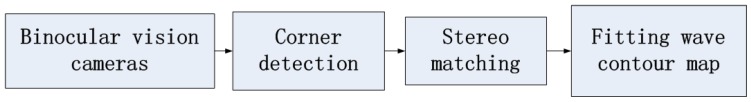
The main steps used by the proposed detection system.

We use binocular cameras to measure the wave height and reconstruct the 3D free surface. To enrich the wave texture, the designed pattern is projected on the water surface. Finally, experiments prove that the proposed measurement approach is effective. The proposed measurement system consists of the following parts: the binocular camera measurement system works externally and is triggered by a signal generator to synchronously capture left and right images. Then, the proposed stereo matching algorithm extracts and matches the features of the left and right images. When drawing the contour map, the camera calibration results, including the fundamental, translation and rotation matrices, are used to calculate the wave height, shown in Figure 1.

### 3.1. Corner Detection

Numerous corner detection algorithms, such as the Harris, Moravec, and Shi–Toamsi corner detection techniques as well as the SIFT algorithm, have been developed for feature extraction. The proposed method is based on the SIFT algorithm in combination with the sub-pixel Harris corner detection algorithm. The SIFT descriptors exhibit good scale-invariant characteristics because of the DOG space; however, the descriptors are at the pixel level, and the numbers of key points are not easy to control. The wave images are sensitive to light, and, thus, it is important that the descriptors can represent the wave textures under different lighting conditions; in addition, it would be convenient if the number of descriptors can be adjusted according to different lighting conditions. Therefore, this paper extracts sub-pixel Harris corners in the DOG space and sets a Harris corner response threshold not only to locate the scale-invariant descriptors more precisely but also to control the number of descriptors that represent the wave texture. Suppose that R(x,y) is the corner response value of corner (x,y), which is the centre of the nearest nine neighbour pixels and can be described by the following second-order polynomial equation:

(1) $$a{x_{i}}^{2}+b{y_{i}}^{2}+cx_{i}y_{i}+dx_{i}+ey_{i}+f=R\left(x_{i},y_{i}\right),$$

where $\left(x_{i},y_{i}\right)$ is the position of the neighbour of (x,y),i∈(1,2,3,‥‥9). These nine equations include six unknowns, namely, a,b,c,d,e and *f*. The sub-pixel position $\left(x^{\prime },y^{\prime }\right)$ is the correct corner position. Hence, substituting the correct position into Equation (1), differentiating and setting the resulting expression equal to zero gives the sub-pixel position $\left(x^{\prime },y^{\prime }\right)$:

(2) $$\left\{\begin{array}{c} x^{\prime }=\frac{2bd-ce}{c^{2}-4ab}\\ y^{\prime }=\frac{2ae-cd}{c^{2}-4ab}. \end{array}\right.$$

**Figure 2:**
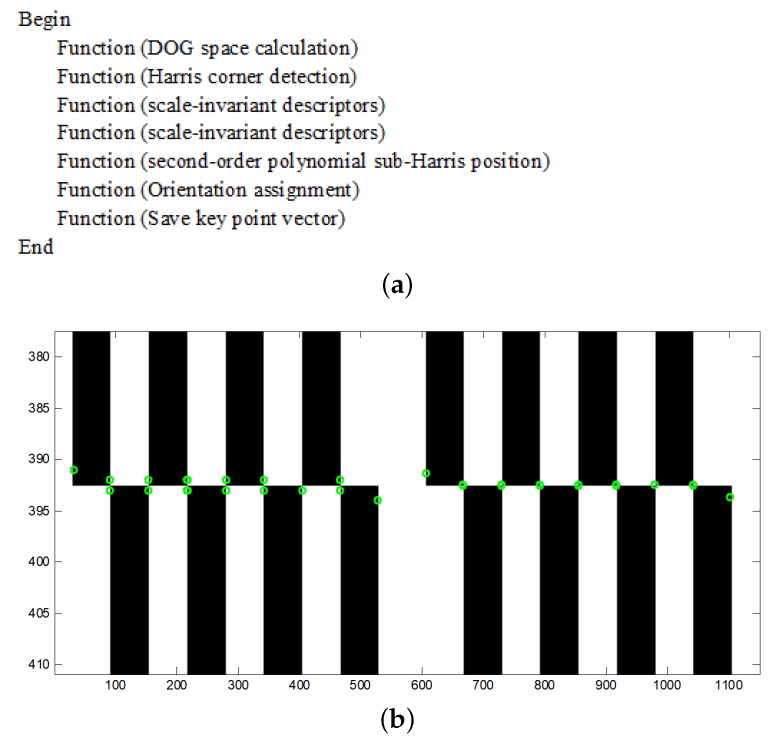
The flowchart and the simulation result. (**a**) the pseudo-code of improved SIFT features extraction; (**b**) to the left of the dotted line is an example of Harris corner detection, whereas an example of sub-pixel Harris corner detection is shown to the right.

Figure 2a shows the pseudo-code of the improved key-point extraction, and Figure 2b shows a comparison between the sub-pixel Harris and Harris corners. Generally, the corners are defined as the intersections of the white and black blocks. In the Harris corner detection panel to the left of the dotted line, two green circles are drawn at each intersection. In contrast, in the sub-Harris corner detection panel to the right of the dotted line, only one green circle is drawn at each intersection, and thus the sub-Harris corners are more accurate than the Harris corner. Along the borders, because the intersections are not obvious, neither algorithm works well.

### 3.2. Stereo Matching Algorithm

**Figure 3:**
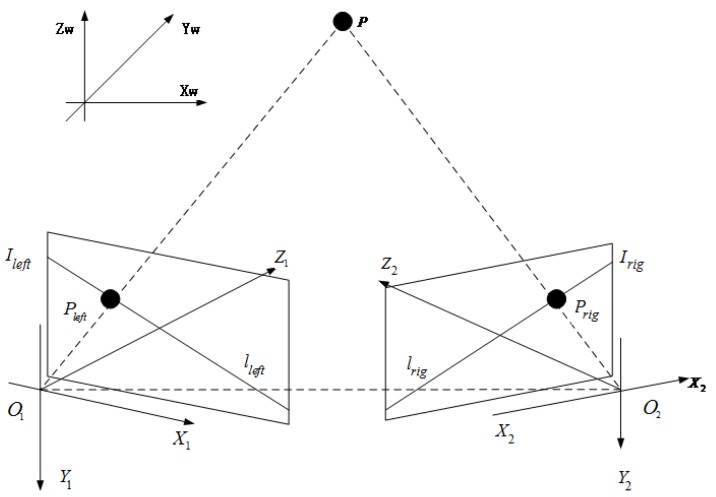
Epipolar constraint.

In this section, a bi-directional matching algorithm is introduced into the SIFT algorithm to decrease the mismatch rate and computation time. In the matching component of the original SIFT algorithm, the Euclidean distance between each corner in the left image and each corner in the right image is calculated, thereby representing a type of two-dimensional searching. However, the binocular camera method can change this process to a one-dimensional search using epipolar constraints (14), shown in Figure 3. In this paper, we propose a bi-directional epipolar constraint. Suppose *P* is a point in space; this point is imaged by the left camera as $P_{left}$ and by the right camera as $P_{rig}$. Therefore, $P_{left}$ and $P_{rig}$ are a pair of correctly matched corners. $I_{left}$ and $I_{rig}$ denote the image planes of the left and right cameras, respectively. The epipolar line is defined as follows:

(3) $$L_{rig}=F\cdot P_{left},$$

(4) $$L_{left}=F^{T}\cdot P_{rig},$$

where *F* is the fundamental matrix. From the definition of the epipolar line constraint, when calculating the candidate matching point of corner $P_{left}$, the epipolar constraint calculates all of the corners along the epipolar line $l_{rig}$ in the right image using the Euclidean distance to judge whether the corner is located on the epipolar line. Let A,B, and *C* be the coefficients of the epipolar line equation, which can then be written into an algebraic expression:

(5) Ax+By+C=0.

**Figure 4:**
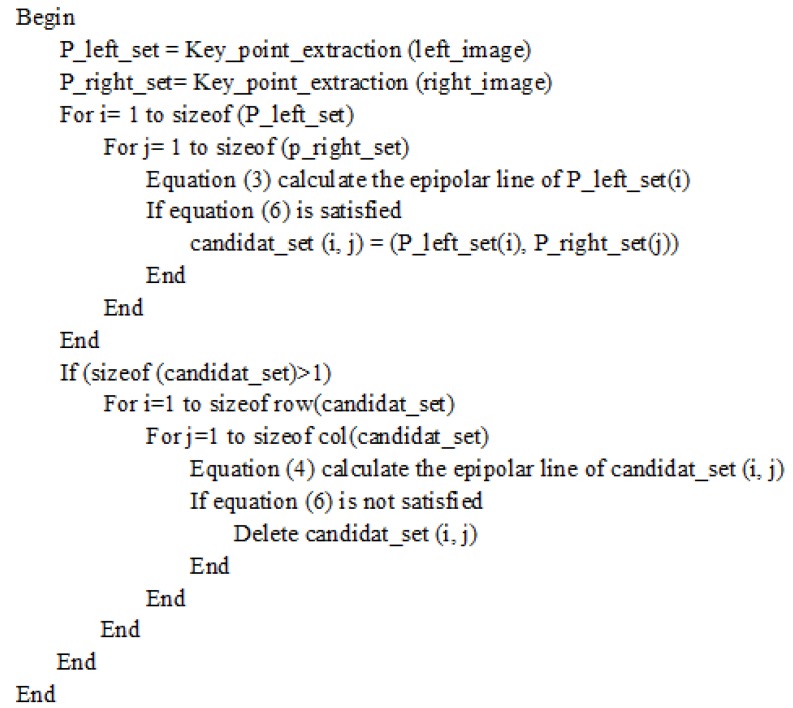
Bi-direction epipolar constraint.

Therefore, a key point in left image calculates the right epipolar line equation by Equation (3). On the right image, all of the key points construct the candidate matching set. In the set, if the distance between the key point to the right epipolar line is less than the threshold *d*, then the key point is the matching point. Suppose that $\left(x^{\prime },y^{\prime }\right)$ is the matching corner in the set, and the distance between $\left(x^{\prime },y^{\prime }\right)$ and the epipolar line must be less than a threshold *d*:

(6) $$\frac{Ax^{\prime }+By^{\prime }+C}{\sqrt{A^{2}+B^{2}}}\leq d,$$

where $\left(x^{\prime },y^{\prime }\right)$ is the matching point. Generally, in the candidate set, more than one key point is always satisfied with Equation (6). Then, each of these key points calculates an epipolar line on the left image using Equation (4). Equation (6) calculates the distances between the $P_{left}$ and these epipolar lines. It must be only in the epiploar line of $P_{rig}$. The other pairs are deleted. The pseudo-code of bi-direction epipolar line is shown in Figure 4.

**Figure 5:**
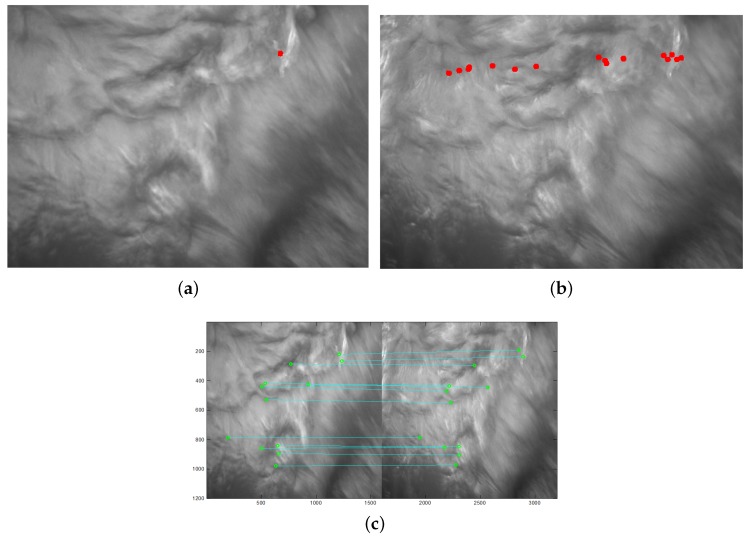
Suppose a corner exists in the left image. When the epipolar constraint is used, the one-to-many phenomenon is generated insomuch that the one corner in the left image matches with 14 corners in the right image. Then, the bi-directional epipolar constraint calculates all of the epipolar constraints of these candidate corners. The correctly matched pair must include the same corner in the left image, that is, the correct pair is defined by the bi-directional epipolar constraint. (**a**) suppose a key point in the left image; (**b**) epipolar constaint in the right image; (**c**) the bi-direction epipolar constraint.

Figure 5 presents an example of the proposed matching algorithm. Figure 5a shows an arbitrary corner in the left image. There are 14 candidate corners in Figure 5b after applying the epipolar constraint due to the one-to-many matching phenomenon. When the bi-directional epipolar constraint is used, a one-to-one match between the left image and right image can be achieved, as shown in Figure 5c. To describe the matched result clearly, the left image and right image are combined and shown in Figure 5c. The horizontal and vertical axis represents the pixel number, and the captured image size is 1628 × 1236. The bi-directional epipolar constraint finds 12 one-to-one match pairs in total.

## 4. Experimental Results

### 4.1. Experimental Equipment

**Figure 6:**
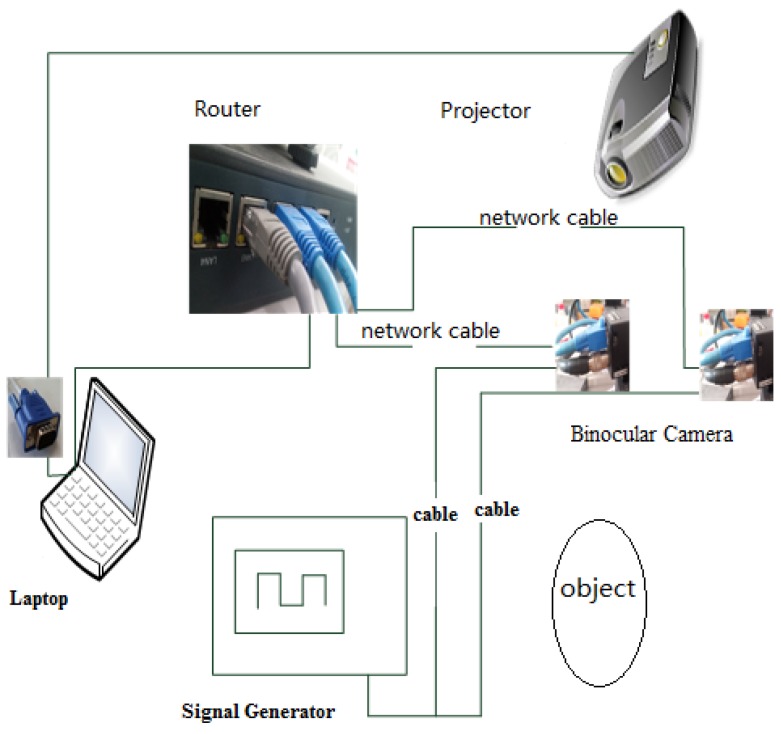
Connections between the individual devices.

**Figure 7:**
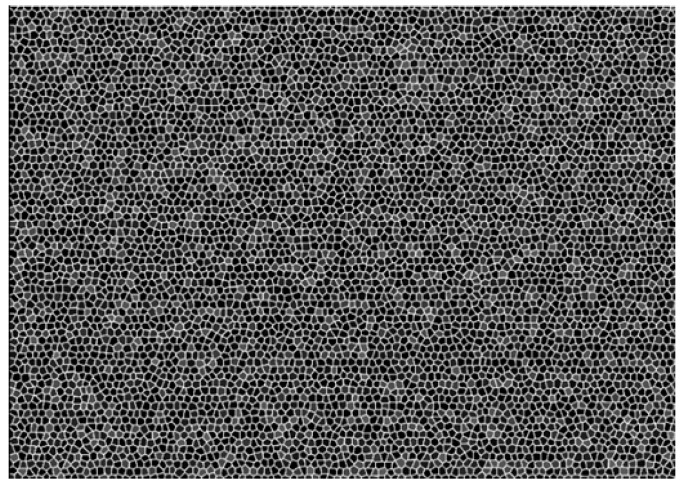
The designed projection pattern.

The experimental equipment includes a binocular camera system, a projector, a signal generator, a set of binocular cameras, a calibration board, a high-speed router, and a main control computer. The connections are shown in Figure 6. MV-VE200SC digital cameras with a gigabit ethernet (GigE) interface are utilized as the binocular cameras. The lenses are Computar M0814-MP2 lenses, and the resolution is 1628 × 1236 at a focal length of 8 mm. The binocular cameras are fixed, and the distance between them is 30 cm. The standard calibration plate is used. Zhang’s calibration algorithm [25] is used to determine the intrinsic and extrinsic parameters of the binocular cameras. It also can calculate the rotation matrix, translation matrix and the fundamental matrix. The binocular cameras are connected to a ThinkPad T440 laptop by a high-speed router. The man–machine interface is written in Visual C++ 6.0 to calibrate and capture the images. A customized characteristic pattern consisting of a black and white hexagonal grid is projected onto the water surface to enhance the water ripple characteristics, shown in Figure 7. To collect pictures synchronously from the binocular cameras and save them automatically, the binocular camera measurement system works in an external trigger mode, where a high-level signal triggers the acquisition of images. The signal generator is connected to the camera by a coaxial cable and generates an external trigger pulse signal with a period of 60 ms, a peak value of 10 V, and a bias of 5 V.

### 4.2. Experiment in a Circular Storage Tank

**Figure 8:**
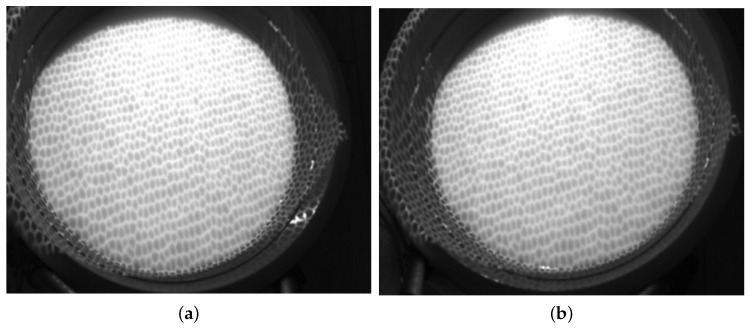
The calm water images. (**a**) the left image; (**b**) the right image.

The water in a 500-mm-wide circular storage tank is dyed to a milky white colour to decrease the penetration of light. The projector projects the black and white hexagonal grid pattern onto the water surface. A cylindrical object with a diameter of 30 mm and a mass of 2 g is allowed to fall freely into the water from above the container to generate micro-scale ripples. A pulse signal generator controls the acquisition of the camera to collect images automatically while the laptop records the wave images during the generation, attenuation and disappearance of ripples in the water storage container. Images of the original calm water are shown in Figure 8.

The correctly matched pairs are used to fit the wave height contour map and draw a three-dimensional mesh figure by employing the least squares (LS) interpolation algorithm. In the calm water experiment, the result of Zhang’s calibration is shown as follows:

(7) $$\begin{array}{c} A_{l}=\left[\begin{array}{ccc} 1957.5 & -10.583 & 845.88\\ 0 & 1953.9 & 603.66\\ 0 & 0 & 1 \end{array}\right], \end{array}$$

(8) $$\begin{array}{c} A_{r}=\left[\begin{array}{ccc} 1898.4 & -0.8175 & 803.89\\ 0 & 1897.5 & 589.58\\ 0 & 0 & 1 \end{array}\right], \end{array}$$

(9) $$\begin{array}{c} T=\left[\begin{array}{ccc} 1898.4 & 14.58 & -0.8686 \end{array}\right], \end{array}$$

(10) $$\begin{array}{c} R=\left[\begin{array}{ccc} 1898.4 & 14.58 & -0.8686\\ 0.0046 & 1 & -0.0032\\ -0.3074 & 0.0045 & 0.9516 \end{array}\right], \end{array}$$

(11) $$\begin{array}{c} F=\left[\begin{array}{ccc} -2.486672e-08 & 2.809117e-07 & 2.586073e-5\\ 1.669562e-06 & -1.4285e-07 & -0.01388912\\ -0.00109649 & 0.01260283 & 1 \end{array}\right], \end{array}$$

(12) $$\begin{array}{c} H_{c}=\left[\begin{array}{cccc} 0.8433 & 0.4749 & 0.2865 & 92.9763\\ 0.5484 & -0.8366 & -0.1158 & 280.9596\\ -0.2035 & 0.2385 & 0.9577 & 783.857 \end{array}\right], \end{array}$$

where $A_{l},A_{r}$ are the intrinsic parameters of the left and right cameras. *R* is the rotation matrix. *T* is the transfer matrix. *F* is the fundamental matrix. $H_{c}$ is the transfer matrix between the binocular cameras coordinate system and the reference coordinate system.

**Figure 9:**
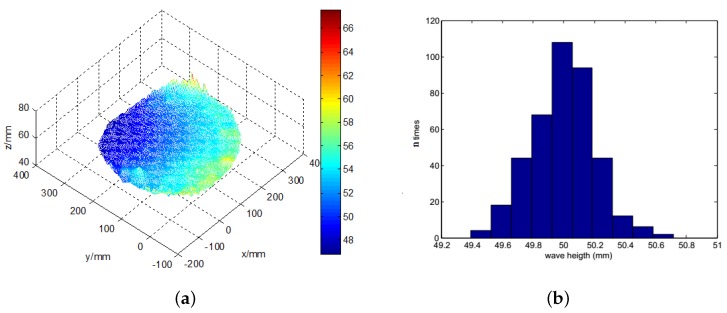
The 3D reconstruction of the calm water surface. (**a**) the measured three dimensional wave; (**b**) the statistics histogram of calm water wave height.

**Figure 10:**
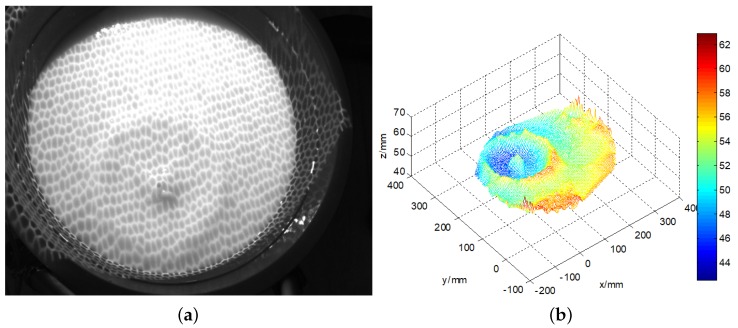
The measured wave height result. (**a**) the original image of the micro-scale wave; (**b**) the measured three-dimensional wave height.

The three-dimensional calm water surface is shown in Figure 9a. The *x*-axis represents the horizontal distance to the left camera, and the *y*-axis represents the vertical distance, while the *z*-axis shows the wave height. The statistics histogram of 400 measurements is shown in Figure 9b, therefore the mean height of calm water is 50 mm. Figure 10a shows the original wave images captured by the binocular cameras. The micro-scale waves generated by the free-falling object are easily read from the images. On the images, we can easily discern a two-level wave; in addition to the centre layer, there is another ripple outside it. In this small container, the wave metre does not work well because of reflected waves. The three-dimensional wave height surface is shown in Figure 10b, which clearly represents the actual wave; even the reflected wave near the edge is also described in detail. The experiments with a calm water surface and micro-scale wave height measurement prove that the binocular camera measurement system can accurately measure the wave height with an absolute error of approximately 2 mm. When measuring the wave height, some influencing factors must be considered. First, in the experiment, because of transformation errors among the different coordinates, the results represent an incline in the detected height of the calm water wave, which is processed as a fixed system error. In sequencing wave height detection, calm water is used as the standard reference plane to increase the detection accuracy. Second, the intensity of the illumination is closely related to the accuracy of the wave height measurement. When the intensity value is excessively high, the wave height cannot be measured where a bright spot is generated. If the intensity value is too low, the entire image will be dark, and the wave height cannot be measured.

### 4.3. Experiment in a Tank

**Figure 11:**
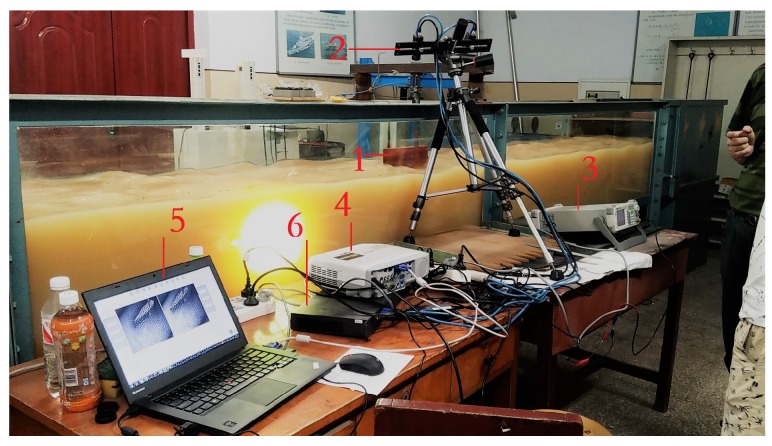
Experimental environment. 1. Airfoil shape; 2. binocular cameras; 3. signal generator; 4. projector; 5. laptop; 6. gigabit Ethernet switch.

Figure 11 illustrates the experimental environment of the tank. The clamp above the circulating water tank fixes the airfoil shape. When the water tank is operating, a wave is generated around the airfoil-shaped model, which is the object being measured. Because the binocular cameras system is not fixed, the calibration is done again. The tank experiment calibration parameters are shown as follows.

(13) $$\begin{array}{c} A_{l}=\left[\begin{array}{ccc} 1921.954 & -0.21298 & 813.4364\\ 0 & 1919.928 & 554.1574\\ 0 & 0 & 1a \end{array}\right], \end{array}$$

(14) $$\begin{array}{c} A_{r}=\left[\begin{array}{ccc} 1805.06 & 0.2540567 & 799.8363\\ 0 & 1904.539 & 580.826\\ 0 & 0 & 1 \end{array}\right], \end{array}$$

(15) $$\begin{array}{c} T=\left[\begin{array}{ccc} 199.6503 & 0.8470257 & 30.097 \end{array}\right], \end{array}$$

(16) $$\begin{array}{c} R=\left[\begin{array}{ccc} 0.9510744 & 0.001605515 & 0.3089486\\ 0.003430925 & 1 & 0.005365272\\ -0.3089337 & 0.006162768 & 0.9510636 \end{array}\right], \end{array}$$

(17) $$\begin{array}{c} F=\left[\begin{array}{ccc} 8.911515e-09 & 7.777782e-07 & -4.896202e-5\\ 9.306116e-06 & -2.794987e-07 & -0.01093195\\ -4.859329 & 0.009531131 & 1 \end{array}\right], \end{array}$$

(18) $$\begin{array}{c} H_{c}=\left[\begin{array}{cccc} 0.7885 & 0.4152 & 0.4584 & -276.167\\ -0.981 & 0.6622 & 0.4487 & -173.113\\ 0.1252 & 0.6324 & 0.7659 & 354.75 \end{array}\right]. \end{array}$$

**Figure 12:**
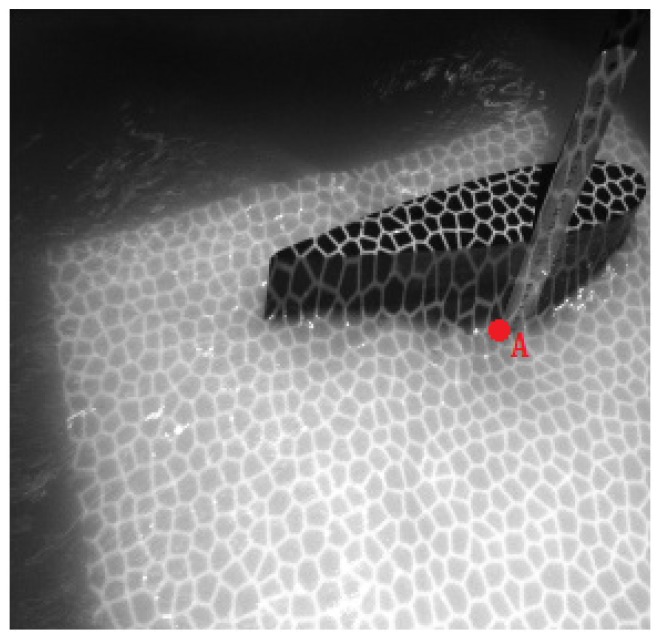
The original image of waves in the tank.

**Figure 13:**
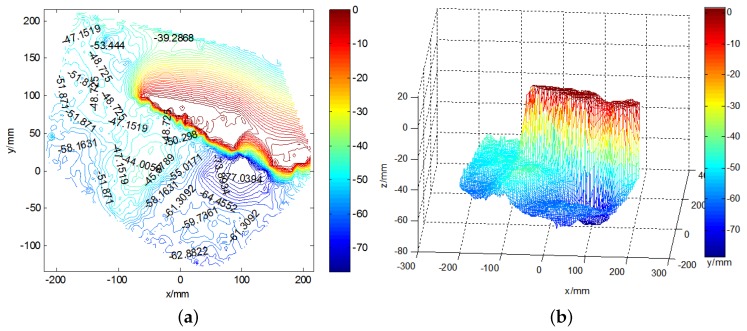
The three-dimensional reconstruction of wave image. (**a**) the wave height contour map; (**b**) the three-dimensional wave height surface figure.

**Figure 14:**
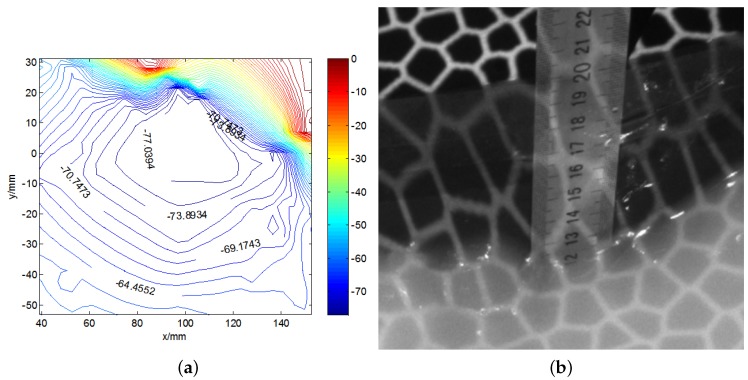
The comparison of the wave height measurement. (**a**) the wave height contour map; (**b**) the Vernier calliper reading.

**Figure 15:**
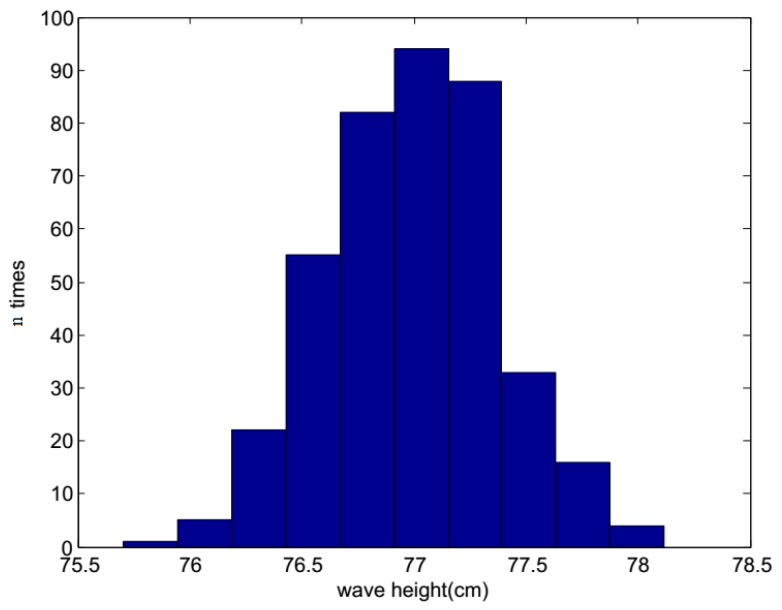
The statistics histogram of calm water wave height.

Figure 12 shows the original images of the waves in the tank, and a pattern is clearly observed. Around the airfoil shape, some micro-scale ripples can be seen. The height ruler is fixed to the side of the airfoil shape, thereby forming a stable standard measurement plane together with the upper surface of the airfoil. The contour map and three-dimensional wave height figure are shown in Figure 13. In Figure 13a, the upper part is the airfoil shape, while the groove in the middle denotes the position of the airfoil support. The bottom part represents the micro-scale wave; in the image, we can read the peaks and troughs, which are not easily recognized in the original wave images. Figure 14a presents the contour map, on which the numbers show the wave height of the discrete scatters. The upper surface height (from the upper surface of airfoil shape to the bottom of the tank is 60 cm) is set as 0; therefore, the wave height value is represented as a negative value. The tank is so small that the wave meter cannot stabilize at a certain value because of the occurrence of strong reflected waves. Although no wave height meter can sufficiently measure the dynamic wave height, this study uses the method used in a PhD thesis [26], in which a Vernier caliper was introduced as the standard measurement, for the sake of comparison with the proposed algorithm, which is shown in Figure 14b. The statistics histogram of 400 measurements of the point A is 77 cm, shown in Figure 15. Because the upper surface height is 60 cm, the wave height of point A is 12 cm, which is in accordance with Figure 13b.

These results prove that the proposed algorithm can measure the dynamic wave height. The contour map displays the wave height over the entire area over time. Some challenges are still encountered in the tank experiment, such as the existence of high-order clutter when the wave is transmitted in the tank. The superposition of clutter introduces some difficulties into the wave height measurement, decreasing the overall accuracy.

## 5. Conclusions

The wave height is an important parameter in coastal ocean dynamics. A binocular camera measurement system can help acquire true contactless measurements. In this study, a binocular camera wave height measurement system is proposed. Based on the SIFT algorithm, sub-pixel Harris corners are detected in the DOG space to locate the key points more accurately. A bi-direction epipolar constraint algorithm is proposed to decrease the mismatch rate and computation time. The experimental results demonstrate that the proposed wave measurement system is suitable for processing water wave images and provides a good foundation for the successive three-dimensional reconstruction of wave contour maps.

References
